# Sequence representations after action-imagery practice of one-finger movements are effector-independent

**DOI:** 10.1007/s00426-022-01645-3

**Published:** 2022-02-03

**Authors:** Stephan Frederic Dahm, Matthias Weigelt, Martina Rieger

**Affiliations:** 1 Institute for Psychology, UMIT-Private University for Health Sciences, Medical Informatics and Technology, Eduard Wallnöfer-Zentrum 1, 6060 Hall in Tyrol, Austria; 2 University of Paderborn, Paderborn, Germany

## Abstract

Action-imagery practice (AIP) is often less effective than action-execution practice (AEP). We investigated whether this is due to a different time course of learning of different types of sequence representations in AIP and AEP. Participants learned to sequentially move with one finger to ten targets, which were visible the whole time. All six sessions started with a test. In the first four sessions, participants performed AIP, AEP, or control-practice (CP). Tests involved the practice sequence, a mirror sequence, and a different sequence, which were performed both with the practice hand and the other (transfer) hand. In AIP and AEP, movement times (MTs) in both hands were significantly shorter in the practice sequence than in the other sequences, indicating sequence-specific learning. In the transfer hand, this indicates effector-independent visual-spatial representations. The time course of the acquisition of effector-independent visual-spatial representations did not significantly differ between AEP and AIP. In AEP (but not in AIP), MTs in the practice sequence were significantly shorter in the practice hand than in the transfer hand, indicating effector-dependent representations. In conclusion, effector-dependent representations were not acquired after extensive AIP, which may be due to the lack of actual feedback. Therefore, AIP may replace AEP to acquire effector-independent visual-spatial representations, but not to acquire effector-dependent representations.

## Introduction

Action-imagery practice (AIP, also mental practice or motor-imagery practice) is the repetitive use of motor imagery with the intention to improve action-execution (Driskell et al., 1994; Simonsmeier et al., 2020). As an alternative to action-execution practice (AEP), AIP has been shown to improve performance in various fields, such as surgery (Arora et al., 2011), music (Coffman, 1990), sports (Guillot et al., 2010), and rehabilitation (Page, 2010). Compared to a non-practice control group, AIP is often followed by performance improvements, although to a lesser degree than AEP (Driskell et al., 1994; Ladda et al., 2021; Simonsmeier et al., 2020; Toth et al., 2020). The present study aims to shed light on the type of representations that lead to performance improvements after AIP.

A key part of AIP is action-imagery, which involves a representation of an action without its actual execution (Jeannerod, 1995). In the present manuscript, we use the term action-imagery rather than motor imagery to emphasize the multimodal nature of imagined actions (Cumming & Eaves, 2018; Dahm, 2020). Action imagery involves not only kinesthetic/motor aspects of the action, but also visual aspects and other modalities of the action, which are related to the action consequences (Dahm, 2020; Kilteni et al., 2018). It is assumed that, at least partly, similar mechanisms take place in action-imagery and action-execution, because similar brain areas are active during both (Lorey et al., 2013; Munzert et al., 2009) and because durations are similarly affected by constraining factors (Dahm & Rieger, 2016a, 2016b; Rieger & Massen, 2014; for an overview see Guillot et al., 2012a, 2012b). However, differences between imagined and executed actions also exist. These differences result from the necessity to inhibit actual action execution in imagination (Guillot et al., 2012a; Rieger et al., 2017). Consequently, no action effects occur on the body and in the environment (Campos et al., 2009). The same holds for AIP in comparison to AEP. Studies on AIP show that AIP and AEP often result in similar changes in brain activation (Pascual-Leone et al., 1995), though this is not always observed (Kraeutner et al., 2020; Nyberg et al., 2006). Behaviorally, AIP leads to performance improvements, albeit to a lesser degree than AEP (Toth et al., 2020). Further, AIP seems to be more effective in cognitive than in motor tasks (Driskell et al., 1994).

Thus, it is imperative to understand what exactly is learned in AIP and how similar the acquired representations in AIP and AEP are (Heuer, 1985). Some evidence indicates that AIP results in partly different action representations than AEP (Amemiya et al., 2010; Frank et al., 2014; Kohl & Roenker, 1989; Kraeutner et al., 2020; Land et al., 2016; Nyberg et al., 2006; Wohldmann et al., 2008). In particular, it seems that AEP results in effector-dependent representations, whereas AIP results in effector-independent representations (Amemiya et al., 2010; Kraeutner et al., 2020; Wohldmann et al., 2007, 2008). Effector-independent (non-motor) representations include representations of an action in visual-spatial coordinates in external space (Imamizu & Shimojo, 1995; Panzer et al., 2009). Effector-dependent mechanisms include motor commands and muscle activation specific to the used effectors (Imamizu & Shimojo, 1995; Panzer et al., 2009). It has been proposed that action representations are more flexible after AIP than after AEP (Wohldmann et al., 2008). One reason for this might be that during AIP no sensory-motor reafferences occur (as no actual action occurs). This may result in more effector-independent representations after AIP than after AEP (Kraeutner et al., 2020).

However, we cannot rule out that it is possible to acquire effector-dependent representations in AIP. Indeed, effector-dependent representations were observed after AIP of an implicit sequence learning task (Ingram et al., 2016). Results from action observation practice further point to this possibility. Although action observation and action imagery differ in some aspects (Holmes & Calmels, 2008; Kim et al., 2017), they both do not involve sensory-motor reafferences which seem to promote the development of effector-dependent representations (Kraeutner et al., 2020). During action observation internal action representations are activated in the observer, similar to those that would be active when the observer performs the action (e.g. Decety & Grèzes, 2006; Jeannerod, 2001, 2006; Wilson & Knoblich, 2005). The observer simulates the action most likely using inverse and forwards models (Blakemore & Decety, 2001). Though, results on action observation practice are contradictory with respect to the kind of representations that are developed during learning. In some instances, effector-dependent representations are acquired (Bird & Heyes, 2005). This indicates that action observation can activate motor representations directly and that motor simulation occurs during action observation practice. Similarly, it is possible, that during AIP a full-fledged simulation of the action, including motor components, occurs (cf. Davidson & Wolpert, 2005; Grush, 2004; Ingram et al., 2019). This implies that it should be possible to acquire effector-dependent representations using AIP (Ingram et al., 2016). Further, the amount of practice seems to play a crucial role for the development of effector-dependent and effector-independent representations. In AEP, there is evidence that during practice effector-independent (spatial) representations develop fast and early, whereas effector-dependent representations develop slower and often only become apparent at later stages of practice (Bapi et al., 2000; Hikosaka et al., 2002; Park & Shea, 2005). However, previous studies of AIP often involved only one single session (Gentili et al., 2010; Land et al., 2016; Shanks & Cameron, 2000), which makes it difficult to investigate the time course of the development of different representations. Thus, under the assumption that mechanisms are partly similar in AIP and AEP, it is possible that in many previous AIP studies (Gentili et al., 2010; Land et al., 2016; Shanks & Cameron, 2000), there has not been enough practice for the formation of effector-dependent representations. It remains therefore unclear whether effector-dependent representations develop too slowly to become apparent in many AIP studies or whether effector-dependent representations are not acquired at all in AIP.

In the present study, we investigated the time course of four different types of representations that can be acquired during learning in AIP and AEP. For this, we used an intermanual transfer paradigm where practice is performed with one hand and (some) tests are performed using the transfer hand (e.g., Senff & Weigelt, 2011; Shea et al., 2011). If performance with the transfer hand improves from pretest to posttest (more than in a control group or a control condition), intermanual transfer has occurred. In particular, we investigated the development of effector-dependent representations, effector-independent intrinsic representations, visual-spatial representations, and abstract representations (see Table 1).

*Effector-dependent representations* reflect kinesthetic and tactile sensations, and involve motor commands, which are specific to the effectors used during practice (Imamizu & Shimojo, 1995; Panzer et al., 2009). Effector-dependent representations were observed for instance in a discrete sequence production task where the MTs in the practice sequence were shorter in the practiced hand configuration than in an unpracticed hand configuration (Verwey & Wright, 2004). In the intermanual transfer paradigm, effector-dependent representations are observable if the practiced action in the practice hand is performed better than both the same action in visual-spatial coordinates in the transfer hand and the mirror action in the transfer hand. Such a comparison of the practice and transfer hand using practice and mirror actions has not been conducted in previous experiments.

*Effector-independent intrinsic representations* involve a body-based reference frame, but are not restricted to the practiced effector. In unimanual actions, ipsilateral and contralateral motor activity occurs, i.e., homologous motor neurons are active. The contralateral activity can be used to facilitate learning of mirror actions (Gordon et al., 1994; Hikosaka et al., 2002). Effector-independent intrinsic representations are observable if practice results in better performance in actions requiring homologous muscles of the transfer hand (i.e., in mirror movements) in comparison to a control group or control actions (e.g., a different sequence).

*Visual-spatial representations* are effector-independent and involve an environment-based (extrinsic, object-centered) reference frame, i.e. they involve visual-spatial coordinates in external space, and are available for any kind of effector (Imamizu & Shimojo, 1995; Panzer et al., 2009). Visual-spatial representations result in better performance of non-mirror actions in the transfer hand in comparison to a control group or a control action.

*Abstract representations* involve information about the structure of a sequence independent from visual-spatial coordinates and effectors (Pothos, 2007). They may for instance involve information about the distance and direction changes between sequence elements. An abstract representation of distance between sequence elements could be ‘long, short, short, long’, which is independent from the effectors that perform the action and the location of the action targets. Abstract representations result in better performance of the mirror action with the practice hand in comparison to a control group or a control action.

The major aim of this study was to investigate, whether representations acquired through AIP and AEP differ per se or whether different representations result from a different time course of acquisition. To the best of our knowledge, the time course of representations acquired during AIP has not been investigated so far. In the present study, an AIP group and an AEP group practiced an explicit sequence, which required pointing movements to multiple targets with one finger (for a similar task see Gentili et al., 2010). The control-practice (CP) group practiced a simpler sequence. Tests involved the practice hand and the transfer hand, each in the practice sequence, a mirror sequence, and a different sequence.

To investigate the time course of learning different representations, participants practiced the task on four separate days. We expected that learning occurs in both AEP and AIP, as sequence learning has been observed in AEP and AIP in previous studies (Ingram et al., 2016; Kraeutner et al., 2016; Land et al., 2016; Wohldmann et al., 2007, 2008). Because it has been shown that effector-independent visual-spatial representations are acquired in AIP (Land et al., 2016) and in AEP (Land et al., 2016; Panzer et al., 2009) and because the task has a strong visual-spatial component, we expected to observe effector-independent visual-spatial representations early in practice, both in AEP and AIP. We further expected that effector-dependent representations are acquired in AEP (Bapi et al., 2000) relatively soon in the course of learning. In AIP, the acquisition of effector-dependent representations should occur later, if it occurs at all (cf. Wohldmann et al., 2008). In the time course of learning, effector-independent intrinsic representations may be acquired in AEP (Panzer et al., 2009) and also in AIP. We had no specific expectations about abstract representations, but assumed that if they are acquired, this would be relatively late in learning.

To investigate intermanual transfer (which provides evidence for effector-independent representations), participants practiced the task with either the left or the right hand. Intermanual transfer has been shown from the dominant to the non-dominant hand, as well as in the reverse direction from the non-dominant hand to the dominant hand (Panzer et al., 2009). However, sometimes transfer is observed only from the dominant to the non-dominant hand, but not vice versa (e.g. Lohse et al., 2010) or the reverse, from the non-dominant to the dominant hand, but not vice versa (e.g. Land et al., 2016; Senff & Weigelt, 2011). Due to a previous study, in which a task roughly similar to ours was used, we expected to observe intermanual transfer from the non-dominant to the dominant hand in AEP and AIP, but not vice versa (Land et al., 2016).

To examine whether knowledge of the sequence differs between AIP and AEP, participants performed a recall and a recognition test. Because participants saw the movement targets continuously during practice and were aware that they always practiced the same sequence, we expected that they are able to recall and recognize the sequence after practice. We did not expect to observe any significant differences between AIP and AEP.

Further, we investigated participants’ focus on kinesthetic and visual elements during practice as this may further influence the type of representations. We expected a stronger focus on visual elements than on kinesthetic elements, because the task required vision to aim for the target keys. Further, the focus on kinesthetic elements may be weaker in AIP than in AEP, because actual kinesthetic feedback is not available during imagery (Dahm & Rieger, 2016b).

To sum up the main hypotheses of the present study (see Table 1 for the respective comparisons): we expected general sequence-unspecific learning and sequence-specific learning after both, AEP and AIP. Effector-independent visual-spatial representations were expected to develop relatively early in AEP and AIP. We further expected that effector-independent intrinsic representations and effector-dependent representations develop later in AIP than in AEP, and that they are correspondingly weaker in AIP than in AEP.

## Methods

### Participants

All participants were right-handed and between 18 and 35 years old. They all reported to have at least a moderate ability to imagine actions clearly and vividly, assessed with the German Version (Dahm et al., 2019) of the Vividness of Movement Imagery Questionnaire (Roberts et al., 2008). Due to technical issues, the data sets of 24 participants were incomplete and therefore not analyzed. Of 160 complete data sets, seven were excluded from analysis due to the following reasons: one participant of the AIP group did not indicate the start of imagination. Three participants of the AEP group performed extremely slow, as indicated by MTs in the first test before practice that were more than 3 standard deviations above the mean of all 160 participants. Removing such outliers is a common procedure (Ilyas & Chu, 2019), as outliers may lead to pretest differences between groups and in consequence bias practice effects. Three participants of the CP group did not always comply with the instructions of the control task. The distribution of sex and the means and standard deviations of age, of the laterality index (assessed with the Edinburgh Handedness Inventory, Oldfield, 1971), and the scores of external visual imagery, internal visual imagery, and kinesthetic imagery (assessed with the Vividness of Movement Imagery Questionnaire: Dahm et al., 2019; Roberts et al., 2008) of the remaining 153 participants are shown in Table 2, separately for each practice group. All participants gave informed consent and the study was approved by the local ethics committee.

The required sample size for the interaction between six groups (the combination of practice group and practice hand) and six test sessions was estimated with G*Power (Faul et al., 2007). We assumed an effect size of *f*= 0.25 and correlations among repeated measures of *r*=0.5. Alpha was set at 0.05 and the power (1-beta) at 0.8 which resulted in a minimum sample size of *N* = 126 (*N*=21 per group).

### Materials

**Fig. 1:**
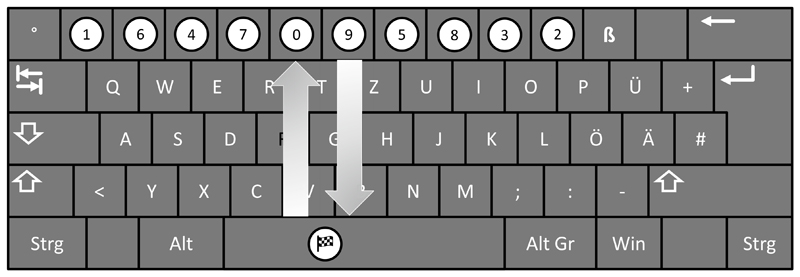
Depiction of sequence A. In the starting position, participants pressed the space bar. Participants were asked to release the space bar, to press the numbered keys from 0 to 9, and to return to the space bar and press it as fast as possible

The experiment was run on participants’ personal notebooks using OpenSesame 3.24 (Mathot Schreij & Theeuwes, 2012). The experiment file is available at https://osf.io/6f9ht/. A white label (diameter = 1 cm) with a checkered flag was put on the space bar indicating the start and end key. Further, labels with black numbers ranging from 0 to 9 (Arial 14) were put on the number keys of the keyboard (see Fig. 1). For the experimental sequences, the numbers on the labels had four possible orders: sequence A (1-6-4-7-0-9-5-8-3-2), sequence A mirrored (AM, 2-3-8-5-9-0-7-4-6-1), sequence B (5-6-3-8-0-9-4-2-1-7), and sequence B mirrored (BM, 7-1-2-4-9-0-8-3-6-5). During the experiment, participants were asked to press the keys according to the numbers in ascending order. Each sequence had the same number of direction changes and involved the same total movement distance. In control sequences, the numbers on the labels were ordered from the left to the right side (C = 0-1-2-3-4-5-6-7-8-9) or from the right to the left side (CM = 9-8-7-6-5-4-3-2-1-0). The task used in the present study has spatial and motor components. It involves both “pure” motor learning, e.g., the optimization of changes of movement direction, and “more abstract” visual-spatial learning, i.e. learning of the target sequence. Note that in this type of sequence learning, the particular order of the keys and corresponding changes in movement direction and the length of the movements are of particular importance, not so much the single key presses themselves.

During the experiment, participants were sometimes asked to change the assignment of the numbers to keys. Each time the labels were adjusted, participants were asked to press the keys according to the numbers on the label with the right thumb, to check whether the assignment of the labels was correct. At this moment, participants had knowledge about the upcoming sequence.

### Task and procedure

**Fig. 2:**
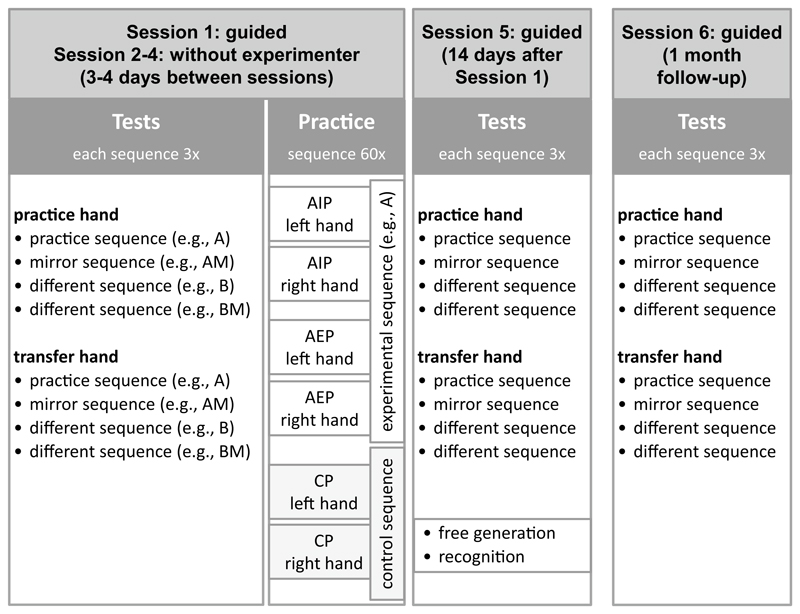
Design of the study. During practice participants performed either action imagery practice (AIP), action execution practice (AEP), or control practice (CP). In the tests, the order of practice hand and transfer hand was counterbalanced across participants

Participants were tested on six testing days (see Fig. 2). To ensure that participants followed the instructions and to answer any questions, an experimenter was present in Session 1, Session 5, and Session 6. In Session 2, 3, and 4, participants practiced at home. The first five sessions were at least two days apart, on average 3.5 days (SD = 0.1 days). Session 6 was performed on average 27.9 days (SD = 2.5 days) after Session 5.

At the start of a trial, participants were asked to press the space bar with the index finger of the hand they were about to use in that trial (starting position). As a starting signal, five red lights (diameter = 2 cm) appeared in intervals of one second, one after another from top to bottom on the screen. The distance between the lights was 0.5 cm. After a random delay between 0.2 and 3 s all five lights turned green. This signaled participants to release the space bar and to start with the sequence. If the space bar was released before the green lights appeared, the starting procedure was repeated. After participants released the space bar, they pressed the numbered keys in the order from 0 to 9 with the index finger, and then returned back to the space bar (Fig. 1). Participants were asked to move as fast and correct as possible.

Each session started with a *familiarization* phase in which participants performed one of the control sequences (counterbalanced across participants) either with the index finger of the left or right hand (counterbalanced across participants). The familiarization sequence was automatically repeated if either (a) reaction time was more than 1500 ms, (b) a wrong key press occurred, or (c) the inter-response-interval (IRI) between the press of the last numbered key and the space bar was more than twice as long as the mean IRIs of the ten previous key presses (0–9).

The familiarization phase was always followed by a test. In the test, each experimental sequence (A, AM, B, and BM) was performed for three trials in a row with each hand. The order of the hands was blocked and counterbalanced across participants. The order of the four sequences was random and the same for each hand. After each trial, participants received feedback about error occurrence and the total movement time in that trial. The feedback was intended to increase participants’ motivation to perform as fast and correct as possible (Wilson et al., 2017).

In the first four sessions, the test was followed by a *practice phase* consisting of 60 trials. For the practice phase, participants were randomly assigned into one of six groups, which were a factorial combination of practice hand (left or right hand) and practice group (action-imagery, action-execution, or control action). In AEP and CP, participants were asked to release the space bar, to move to the numbered keys to press them, and to press the space bar again at the end of a trial. In AIP, participants were asked to release the space bar, to imagine moving to the numbered keys and pressing them, and to actually press the space bar at the moment they imagined getting back to it. In AEP and AIP, one of the experimental sequences was practiced. In control-practice, participants practiced one of the control sequences. After each trial, all participants received feedback about the total movement time in that trial. Every ten trials, a short rest of 5 s was enforced. Each practice phase took between 15 to 20 min, as suggested by previous research (Driskell et al., 1994; Toth et al., 2020).

After the practice phase in Session 4, participants indicated how strongly they felt/imagined to feel and saw/imagined to see how their fingers touched the keys on rating scales (from 1—‘not at all’ to 9—‘very strongly’).

In Session 5, after the test, participants performed a *free generation* test. For the free generation test, participants put empty white labels on the number keys. Afterwards, they were asked to recall and execute the practice sequence using the practice hand. This was followed by a *recognition* test, in which participants performed each of the experimental sequences once with the practice hand. After each sequence, they rated whether it had been the practice sequence from 1—“very unlikely” to 9—“very likely”. The order of the four sequences in the recognition test was randomized without restrictions. In Session 6, participants performed the familiarization phase and the test phase.

### Data analysis

Movement time (MT) was defined as the time between the release of the space bar at the start of a trial and the press of the space bar at the end of a trial. During the tests all error rates were below 10%.

In each test, the four experimental sequences were each performed three times with each hand (Fig. 2). For the analysis of the tests, median MTs were calculated from the correct trials for each sequence per hand. Overall, 95.5% of all trials were performed correctly.^1^ MTs of incorrect trials were excluded from analysis, because errors (e.g., omissions, insertions, alternations of responses) may have influenced movement duration and (not) detecting errors may have prolonged the completion of the ten responses. The AEP and AIP group practiced one of the four tested sequences, which is called the practice sequence. Another of these sequences was the mirror sequence of the practice sequence. The other two sequences were different to the practice sequence, with one of them being the mirror sequence of the other. This was done to have an equal number of each sequence and its mirror sequence. In AIP and AEP, we randomly chose one of the two different sequences for analysis (to obtain equal reliability across practice, mirror, and different sequences). In CP, the tested sequences all differed from the practiced control sequence. Therefore, three of the four tested sequences were randomly assigned to the practice, mirror, and different sequence condition. We also analyzed movement times during practice. For this, median MTs were calculated from the sixty trials of each session. For the analysis during practice, incorrect trials were included because they cannot be detected in AIP. To analyze recall performance in the free generation test, we calculated the number of triplets that matched with the practice sequence and, as a control, with the mirror sequence (Bird & Heyes, 2005). Here, a score of 8 indicates a full match and a score of 0 indicates no match of the sequences. We further analyzed sequence recognition ratings and the scores of the strength of visual and kinesthetic representations during practice.

Dependent variables were analyzed using mixed model ANOVAs. If Mauchly’s test indicated that the assumption of sphericity was violated, we report Huyn-Feld corrected degrees of freedom and p-values. Further comparisons were conducted using *t* tests with Sidak adjusted pairwise comparisons. Where appropriate, we report minimum (*p*_min_) or maximum (*p*_max_) statistical values. Statistical significance was set at *p* < 0.05. Raw data as well as the syntax for data preparation and data analyses are available at https://osf.io/6f9ht/.

## Results

### Movement times in test blocks

At first, we conducted an ANOVA with the between factors practice group (AIP, AEP, CP) and practice hand (left, right) and the within factors test hand (practice, transfer), sequence (practice, mirror, different), and session (1, 2, 3, 4, 5, 6) on MTs. A significant interaction between practice hand and test hand, *F*(1, 147) = 68.9, *p* < 0.001, $\eta_{\text{p}}^{2}=0.32$, revealed that MTs were significantly shorter in the right hand (which is the practice hand for the right hand practice group and the transfer hand for the left hand practice group) than in the left hand (which is the practice hand for the left hand practice group and the transfer hand for the right hand practice group). However, no further effects involving the factor practice hand were significant (maximum $\eta_{\text{p}}^{2}=0.02$).

**Fig. 3:**
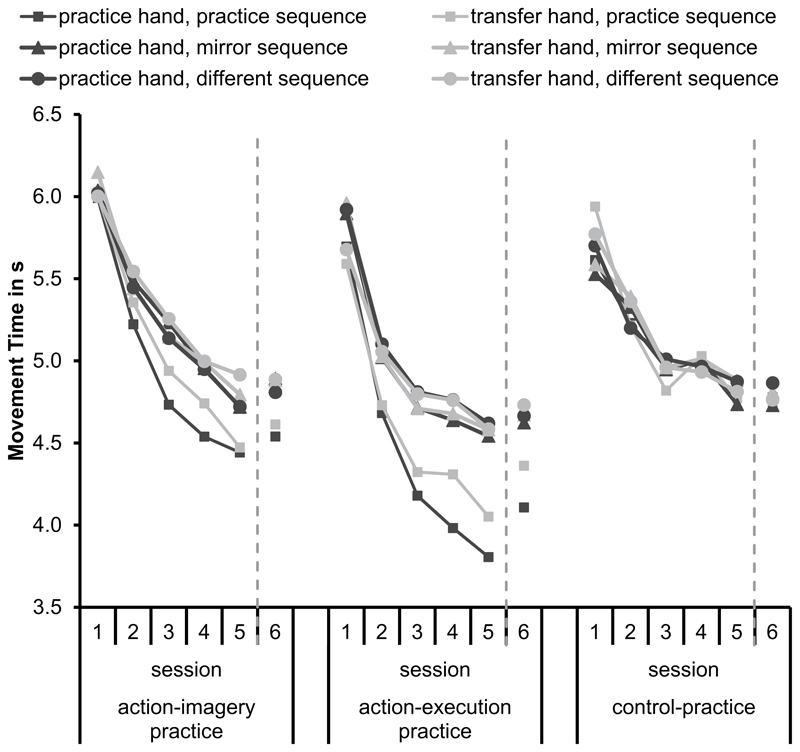
Means of movement times depending on hand (practice, transfer), sequence (practice, mirror, different), and session (1, 2, 3, 4, 5, 6) separately for the practice groups (action-imagery, action-execution, and control action)

To increase readability of the manuscript, we decided to average data over the factor practice hand for the analyses we report here.^2^ Means of MTs are shown in Fig. 3. Standard errors are shown in the supplemental material. We conducted a mixed model ANOVA with the between factor practice group (AIP, AEP, CP) and the within factors hand (practice, transfer), sequence (practice, mirror, different), and session (1, 2, 3, 4, 5, 6) on MTs. Results of the ANOVA are shown in Table 3. For an overview of the relevant comparisons on the research questions see Table 1.

The significant main effect session indicated that MTs became significantly shorter over sessions in all groups. Specifically, MTs became significantly shorter from Session 1 to Session 2 (*p* < 0.001) and from Session 2 to Session 3 (*p* < 0.001). No significant difference in MTs was observed between Session 3 and Session 4 (*p* = 0.31). Further, MTs became significantly shorter from Session 4 to Session 5 (*p* < 0.001), but no significant difference in MTs was observed between Session 5 and Session 6 (*p* = 0.48). These effects provide evidence for sequence-unspecific learning, because they occurred in all groups and all sequences.

Differences in sequence-specific learning between the practice groups were observed in the significant interaction between practice group and session, the significant interaction between practice group and sequence, and the significant interaction between practice group, session, and sequence. In Session 1, MTs did not significantly differ between the groups in all sequences (*p*_min_ = 0.062). Further, in AEP and AIP, MTs did not significantly differ between sequences in Session 1 (*p*_min_ = 0.075), except for longer MTs in the mirror sequence than in the practice sequence in the practice hand in AEP (*p* = 0.044). Hence, performance did not differ significantly between groups and sequences before practice started. In the following sessions, MTs of the mirror and different sequence did not significantly differ between the groups (*p*_min_ = 0.079), except for significantly shorter MTs of the mirror sequence in AEP than in AIP in Session 3 (*p* = 0.031). From Session 2 to Session 5, MTs in the practice sequence were significantly shorter in AEP than in AIP (*p*_max_ = 0.025) and CP (*p*_max_ = 0.009). In Session 6, MTs in the practice sequence did not significantly differ between AEP and AIP (*p* = 0.091), but were significantly shorter in AEP than in CP (*p* = 0.003). MTs in the practice sequence did not significantly differ between AIP and CP in all sessions (*p*_min_ = 0.087). These effects provide evidence for stronger sequence-specific learning in AEP than in AIP.

The significant main effect sequence was modified by the significant interactions between practice group and sequence, between session and sequence, and between hand and sequence. In CP, MTs did not significantly differ between sequences in both hands and all sessions (*p*_min_ = 0.078). This was expected: as the CP group did not learn any of the tested sequences, no sequence specific-learning can occur.

In AEP in both hands, MTs were significantly shorter in the practice sequence than in the other sequences from Session 2 onwards (*p*_max_ = 0.007). This indicates that effector-independent visual-spatial representations were acquired. Further, MTs in the practice sequence were significantly shorter in the practice hand than in the transfer hand (*p* = 0.014) indicating the acquisition of effector-dependent representations in AEP.

In AIP in the practice hand, MTs were significantly shorter in the practice sequence than in the other sequences from Session 2 onwards (*p*_max_ = 0.028). In AIP in the transfer hand, MTs in the practice sequence were significantly shorter than in the different sequence from Session 3 onwards (*p*_max_ = 0.013) and were significantly shorter than in the mirror sequence from Session 4 onwards (*p*_max_ = 0.024). As in AEP, this indicates effector-independent visual-spatial representations. MTs in the practice sequence did not significantly differ between practice and transfer hand (*p*=0.087). Thus, there was no evidence for effector-dependent representations in AIP.

### Movement times during practice

**Fig. 4:**
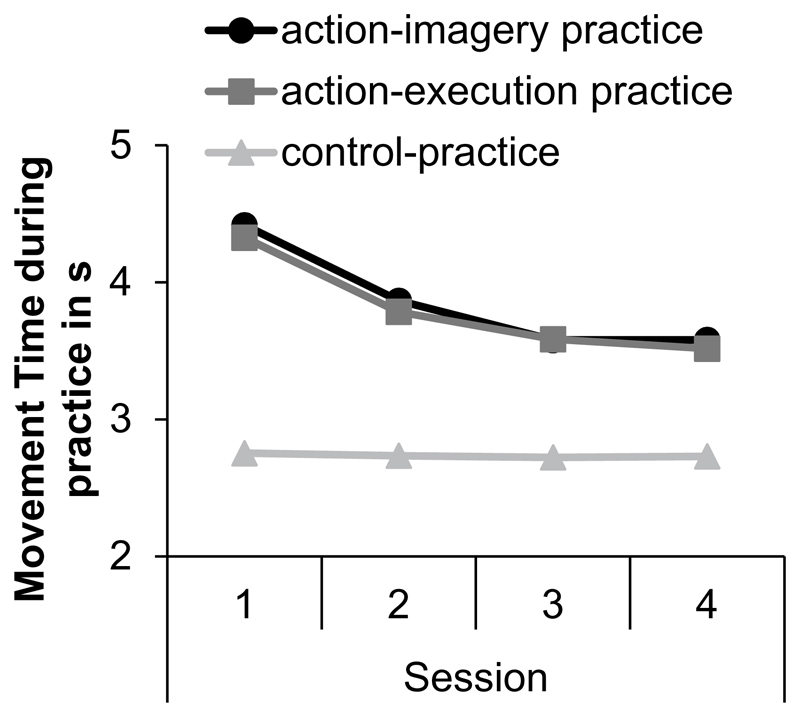
Means of the movement time during the practice sessions (1, 2, 3, 4) in the action-imagery practice, action-execution practice, and control-practice groups

To explore whether MTs became shorter during practice, we calculated the median of the sixty practice trials of each session. Means of MTs during the practice sessions are shown in Fig. 4. Standard errors are shown in the supplemental material. An ANOVA with the between factor practice group (AIP, AEP, CP) and the within factor session (1, 2, 3, 4) was conducted on MTs.

The significant main effect of practice group, *F* (2, 150) = 36, *p* < 0.001, $\eta_{\text{p}}^{2}=0.33$, indicated that MTs were shorter during CP (*M* = 2.7 s, SE = 1.1 s) than during AIP (*M* = 3.9 s, SE = 1.1 s, *p* < 0.001) and AEP (*M* = 3.8 s, SE = 1 s, *p* < 0.001). MTs in AIP and AEP did not significantly differ from each other (*p* = 0.97). The significant main effect session, *F* (2, 299.5) = 72.7, *p* < 0.001, $\eta_{\text{p}}^{2}=0.33$, was modified by the significant interaction between practice group and session, *F* (4, 299.5) = 16.3, *p*< 0.001, $\eta_{\text{p}}^{2}=0.18$. In CP, MTs did not significantly differ between sessions (*p*_min_ > 0.99). In AEP and AIP, MTs became significantly shorter from Session 1 to Session 2 (*p*_max_ = 0.001) and from Session 2 to Session 3 (*p*_max_ = 0.001), but did not significantly differ between Session 3 and Session 4 (*p*_min_ = 0.71).

### Recall and recognition tests

To analyze recall performance, a mixed model ANOVA with the between factor practice group (AIP, AEP, CP) and the within factor sequence (practice, mirror) was calculated on the number of matching triplets. The significant main effect practice group, *F* (2, 150) = 67.3, *p* < 0.001, $\eta_{\text{p}}^{2}=0.47$, and the significant main effect of sequence, *F* (1, 150) = 39.3, *p* < 0.001, $\eta_{\text{p}}^{2}=0.21$, were modified by the significant interaction between practice group and sequence, *F* (2, 150) = 32, *p* < 0.001, $\eta_{\text{p}}^{2}=0.3$. In the CP group, the recalled sequence showed significantly more matches with the practiced control sequence (*M* = 5.7, SE = 0.4) than with the mirrored control sequence (*M* = 0.7, SE = 0.3, *p* < 0.001). However, matches with the recalled sequence did not significantly differ between practice and mirror sequence in the AIP group (practice: *M* = 0.4, SE = 0.4; mirror: *M* = 0.5, SE = 0.3 *p* = 0.91) and AEP group (practice: *M* =0.9, SE = 0.3; mirror: *M* = 0.5, SE = 0.3, *p* = 0.48).

To analyze recognition performance, a mixed model ANOVA with the between factor practice group (AIP, AEP, CP) and the within factor sequence (practice, mirror, and different) was calculated on the rating that a previously performed sequence corresponded with the practice sequence (note that only the experimental sequences, not the control sequence, were used in this test). The significant main effect practice group, *F* (2, 150) = 27.5, *p* < 0.001, $\eta_{\text{p}}^{2}=0.27$, indicated that recognition ratings were significantly lower in the CP group (*M* = 3, SE = 0.3) than in the AIP group (*M* = 5.2, SE = 0.3, *p* < 0.001) and AEP group (*M* = 5.8, SE = 0.3, *p* < 0.001). Ratings in the AIP group and AEP group did not significantly differ from each other (*p* = 0.27). The significant main effect sequence, *F* (2, 300) = 6.1, *p* = 0.003, $\eta_{\text{p}}^{2}=0.04$, indicated that ratings were higher for the practice sequence (*M* = 5.1, SE = 0.2) than for the mirror sequence (*M* = 4.4, *SE* = 0.2, *p* = 0.011) and the different sequence (*M* = 4.5, SE = 0.2, *p* = 0.017). The ratings for the different sequence did not significantly differ from the ratings for the mirror sequence (*p* = 0.97). The interaction between practice group and sequence, *F* (4, 300) = 1.8, *p*=0.14, $\eta_{\text{p}}^{2}=0.02$, was not significant.

### Strength of representation during practice

**Fig. 5:**
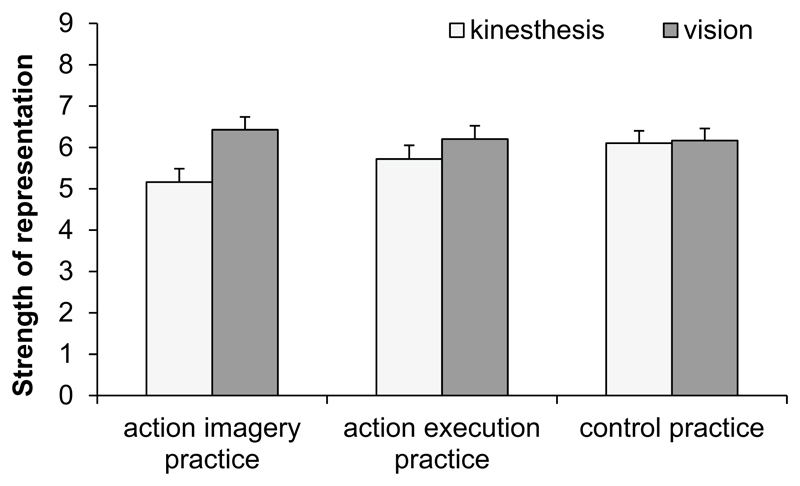
Means and standard errors of strength of kinesthetic and visual representations in the action-imagery practice group, action-execution practice group, and control-practice group

To explore whether participants focused on the same content in AEP and AIP, we analyzed the questions on the strength of kinesthetic and visual representation after the last practice block in Session 4. Means and standard errors of kinesthetic and visual representations are shown in Fig. 5. An ANOVA with the between factor practice group (AIP, AEP, CP) and the within factor modality (kinesthesis, vision) was conducted.

The significant main effect of modality, *F* (1, 148) = 10.2, *p* = 0.002, $\eta_{\text{p}}^{2}=0.06$, was modified by the significant interaction between practice and modality, *F* (2, 148) = 3.4, *p* = 0.037, $\eta_{\text{p}}^{2}=0.04$. The representation of vision was stronger than the representation of kinesthesis in AIP (*p* < 0.001), but not in AEP (*p* = 0.13) and CP (*p* = 0.85). The main effect practice was not significant, *F* < 1.

## Discussion

The aim of the present study was to investigate the acquisition of effector-dependent representations, effector-independent intrinsic representations, visual-spatial representations, and abstract representations in AIP. For this, participants learned an explicit sequence, which required one-finger movements to multiple targets over several sessions using AIP, AEP, or CP. Tests consisted of the practice sequence, a mirror sequence, and a different sequence, each in the practice and transfer hand. In all groups, MTs became shorter from session to session, indicating sequence-unspecific learning. Further, sequence-specific learning was observed. MTs became shorter in the practice sequence than in the other sequences. Because this was observed not only in the practice hand, but also in the transfer hand, it indicated effector-independent visual-spatial representations in both, the AIP and AEP group. Sequence-specific learning was stronger in AEP than in AIP in the practice hand, but not in the transfer hand, indicating stronger effector-dependent representations in AEP than in AIP. The time course of learning did not significantly differ between AIP and AEP.

### Sequence-unspecific learning

MTs in the tests became successively shorter in all groups during the first three sessions. Such sequence-unspecific general learning has been observed previously in AIP and AEP (Wohldmann et al., 2008). Sequence-unspecific learning reflects that participants adapt to the task requirements. For instance, they may learn to move their index finger in a more structured way, such that each button press is performed faster. In the present study, such sequence-unspecific learning can be clearly dissociated from sequence-specific learning, because it also occurred in the tests in CP which did not involve practicing the experimental sequences.

### Sequence-specific learning

It was our primary aim to examine the type of representation learned in AIP. In AEP and AIP from Session 2 onwards, MTs in the practice hand were shorter in the practice sequence than in the different sequence. This indicates that representations of the sequence were acquired. AIP has already been shown to promote the acquisition of representations of a sequence in previous studies (Kraeutner et al., 2016; Land et al., 2016; Wohldmann et al., 2007, 2008). For instance, mentally practiced 4-digit numbers were typed faster than unpracticed 4-digit numbers (Wohldmann et al., 2007). In the following, we will discuss the type of the acquired representations.

*Effector-independent intrinsic representations* of a sequence are observed by better performance in mirror actions of the transfer hand in practice groups than in a control group (Bird & Heyes, 2005; Gruetzmacher et al., 2011; Hayes et al., 2012; Land et al., 2016) or by better performance of mirror actions than of control actions in the transfer hand (Bird & Heyes, 2005). However, neither of this was observed in the present study. Possibly, the present task did not require enough motor coordination to result in the development of effector-independent intrinsic representations. This result is in contrast to Land et al. (2016), who observed better performance in mirror actions of the transfer hand after video-based AIP and AEP than in a no-practice control group. These differences in results might be explained by procedural differences. In the study of Land et al. (2016) several fingers were used to perform the sequence, whereas in the present study the sequence was performed using only the index finger. Other studies that observed intrinsic representations in preplanned action sequences used extension-flexion movements of the fore-arm (Gruetzmacher et al., 2011; Kovacs et al., 2009, 2010). In comparison, the present sequences involved more complicated visual elements and participants may have searched for the position of each target key, even though the target keys were visible before action initiation. This may have promoted visual-spatial representations rather than intrinsic representations. In AEP, ratings on strength of representation indicated that the focus on kinesthetic and visual aspects was equal in the present task. Possibly, effector-independent intrinsic representations of a sequence are built only in tasks that involve more kinesthetic than visual elements. It might also be that mirror transfer has been observed in previous studies (e.g., Land et al., 2016), because parts of the action elements were equal in the practice (index, middle, ring, ring, middle, index finger) and mirror sequence (ring, middle, index, index, middle, ring finger). Similar to our study, little or no transfer to a mirror action of the unpracticed hand was observed in a maze tracking task (van Mier & Petersen, 2006). No transfer to a mirror action implies that the acquired representations in AIP and AEP were not intrinsic in nature, even after extensive practice.

*Visual-spatial representations* are observed if performance in the transfer hand is better in the practice sequence than performance in a different sequence or in a control group. We observed shorter MTs in the practice sequence than in the other sequences in the transfer hand in AEP and in AIP. Hence, effector-independent visual-spatial representations were acquired in AIP and AEP, which is in line with previous results (Ingram et al., 2016; Wohldmann et al., 2008). Further, in the practice sequence of the transfer hand, MTs were shorter in AEP than in AIP and CP. However, MTs in the practice sequence did not significantly differ between AIP and CP. This may be the case because within-participant effects are easier to detect than between-participant effects.

*Effector-dependent representations* are observed if performance improves more in the practice hand than in the transfer hand (Hikosaka et al., 2002). In AIP and AEP, MTs in the practice sequence were shorter than MTs in the other sequences. This effect was more pronounced in the practice hand than in the transfer hand in AEP, but not in AIP. Hence, effector-dependent sequence learning occurred in AEP, but not in AIP. This is in line with previous results showing stronger effector-dependent representations after AEP than after AIP (Land et al., 2016). One explanation is that actual kinesthetic feedback is important for the acquisition of effector-dependent representations (Ingram et al., 2019). In our study, this is further supported by the observation that the strength of kinesthetic representations was lower than the strength of visual representations in AIP, but not in AEP. Although kinesthetic feedback can be imagined, it may not provide sufficient information to detect all performance errors (Dahm & Rieger, 2019a, 2019b; Rieger et al., 2011) to optimize effector-dependent representations.

*Abstract representations* could have been observed if performance in the practice hand was better in the mirror sequence than in the different sequence. In the present study, abstract representations could involve the representation of distance and direction changes between sequence elements. However, this was not the case. Hence, sequence representations did not involve information about the structure independent from visual-spatial coordinates and effectors (Pothos, 2007).

We had expected that effector-independent visual-spatial representations (observable in the practice sequence in the transfer hand) evolve at the beginning of learning and that effector-independent intrinsic representations (observable in the mirror sequence in the transfer hand) evolve at later stages of learning (Panzer et al., 2009). However, the mirror sequence did not significantly differ from the different sequence at any stage of learning in both AIP and AEP. Although MTs during practice indicated a learning plateau after three days of practice, representations may still change. Therefore, one might argue that the present study did not involve enough practice for effector-independent intrinsic representations to develop. Alternatively, and more likely, the absence of effector-independent intrinsic representations in both AIP and AEP may be due to task characteristics. The present task involved many visual-spatial elements, which may have promoted effector-independent visual-spatial representations (see also Wohldmann et al., 2007, 2008).

### Follow-up performance

Performance in the follow-up test indicated that sequence-specific and sequence-unspecific performance enhancements were retained after four weeks without further practice in both hands. Such long-term performance enhancements are sometimes regarded as a better indicator for learning than performance enhancements shortly after training, which may be transient (Kantak & Winstein, 2012). Interestingly, long-term performance enhancements were observed in both AEP and AIP. MTs did not significantly differ between Session 5 and Session 6. Further, in the follow-up test, MTs were shorter in the practice sequence than in the different sequence after AEP and AIP. This indicates that the acquired visual-spatial representations remain stable for several weeks. This is in line with previous studies indicating stable effector-independent visual-spatial representations after AIP (Wohldmann et al., 2007, 2008) and contradicts the assertion that AIP effects are not as robust as AEP effects (Driskell et al., 1994).

### Recall and recognition performance

Whereas several participants of the CP group were able to recall the control sequence three days after the last practice session, participants of the AIP and AEP groups were not able to recall the practice sequence. However, in the recognition test, participants were able to differentiate the practice sequence from the mirror sequence and different sequences. Hence, they were able to recognize at least parts of the sequence. It is surprising that participants’ recall performance was not better, because they were able to see the complete sequence during practice. This result further stands in contrast to results showing that participants can (at least partly) recall an implicitly learned six-element sequence after action observation practice (Bird & Heyes, 2005). Explanations for the present results may be that the sequence was relatively long (10 key presses) and that the sequence was always visible during practice, which makes it unnecessary to memorize it. Most importantly, for the present study, recall and recognition performance did not significantly differ between AIP and AEP. Hence, sequence knowledge is acquired similarly in AIP and AEP and the results concerning partly different representations in AIP and AEP cannot be explained by sequence knowledge.

### Direction of intermanual transfer

In the present study, MTs were not significantly influenced by the direction of intermanual transfer, which is an indication of symmetric intermanual transfer. This is in line with results showing that intermanual transfer does not significantly depend on whether practice occurs with the left or right hand if timing parameters are assessed (Pan & Van Gemmert, 2013b), which we did in the present study. Symmetric transfer has for instance been observed in sequential elbow flexions and extensions (Panzer et al., 2009), finger tapping (Koeneke et al., 2009), a pegboard task (Schulze et al., 2002), and maze tracing (van Mier & Petersen, 2006). Asymmetric intermanual transfer may be more frequent in tasks in which action initiation (Pan & Van Gemmert, 2013a, 2013b) or endpoint accuracy (Sainburg & Wang, 2002; Senff & Weigelt, 2011; Wang & Sainburg, 2006) is measured.

### Limitations

One limitation of the present study is that learning may not only have occurred during practice, but also during tests. This limitation applies to all studies that have a pretest–post-test design, but may be a particular issue in the present study, because participants were tested in each of the six sessions. Therefore, AIP in the present study may not be pure AIP, but a combination of AEP and AIP. In the AIP group, the ratio of execution to imagination of the practice sequence was 3/60. To preempt this limitation, some studies skipped pretesting (e.g., Kraeutner et al., 2016). In the present study, several tests were indispensable to investigate the time course of learning. However, even though learning during tests may be responsible for sequence-unspecific learning effects that took place in all groups, it cannot account for sequence-specific learning, because all sequences were tested equally often.

In the present study, the control group was not a waiting control group, but an active control group, which practiced a simpler sequence. Active control groups are considered more conservative than waiting control groups, as they can overcome differences in expectations caused by placebo-effects (Boot et al., 2013). In our study, control practice benefited ‘general sequence-unspecific learning’ related to the task environment and pressing keys in a sequential order. However, control practice did not benefit sequence-specific learning.

One may argue that participants may not always have adhered to the instruction to imagine the action during AIP. This cannot be directly controlled in AIP, because no actual actions take place (Dahm, 2020). However, no significant differences were observed in the focus on kinesthetic and visual aspects during practice between AIP, AEP, and CP. This indicates that task engagement was probably similar. Further, by logging MTs during AIP, we were able to check for reasonable timing during practice. Timing in MTs during practice was reasonable and the data patterns were similar in AIP and AEP, i.e. they became shorter over the course of learning. Most importantly, our results show sequence-specific learning effects in AIP. Thus, we assume that most participants (if not all) adhered to the instructions during AIP.

### Perspectives

Are the present results generalizable to more complex actions that require the coordination of the whole body? The requirement to learn an action route that involves a particular order of long and short turns, as in our study, is also a characteristic of whole-body actions, for instance in sports. For example, skiing requires performing turns. In a ski run, gates are passed in a particular order. Similarly, in motorbike racing (e.g., MotoGP), a particular sequence of whole-body actions is required to pass the racing circuit repeatedly. We speculate that in those (and other) scenarios, professionals may benefit from performing AIP to acquire visual-spatial representations of a specific route or a specific racing circuit. Further, the performance of complex actions that require sequential steps in a particular order with a specific timing, like a somersault in gymnastics, may also benefit from visual-spatial representations acquired in AIP (Holmes & Collins, 2001).

The observation that visual-spatial representations were acquired might be due to task characteristics (e.g., the use of visual targets), rather than an inherent characteristic of AIP. In everyday life, not all actions have predefined visible targets. For instance, juggling or playing the violin requires performing a predefined sequence of action elements, but the target positions are not visually accentuated. Instead, the actor might rely more on kinesthetic information. Even in motorbike racing and skiing where the predefined targets of the sequence are visible, the targets do not correspond to a single action element. Further, performance may require a stronger focus on kinesthesis to update information about the body position in external space. A stronger focus on kinesthetic information processing than on vision might more strongly support the acquisition of intrinsic representations as observed in sequential waveform drawing (Panzer et al., 2009), figure drawing (Thut et al., 1996), two-ball rotations (Reissig et al., 2015), and finger-to-thumb sequences (Dickins et al., 2015; Land et al., 2016). Future studies may therefore investigate other tasks to investigate the acquisition of intrinsic representations in AIP.

From an applied point of view, the present study indicates that the acquired type of representation differs between AIP and AEP. Hence, AIP is not only a helpful tool that can be used instead of AEP when AEP is not possible (for instance, practicing skiing when there is no snow or when one has an injury), but promotes the learning of specific types of action representation. Because visual-spatial representations are considered more important in the beginning of learning, AIP is assumed to facilitate learning particularly if it is placed in advance to AEP (Kraeutner et al., 2020). However, minimal experience in execution may be helpful to imagine the action appropriately (Decety, 1991; Mulder et al., 2004; Olsson & Nyberg, 2010). Additionally, future studies may consider investigating learning of different representation types in AIP in relearning situations. For instance, when the learner has developed effector-dependent representations for one action and then learns a slightly different action that needs to be adjusted.

## Conclusion

During practice of sequential one-finger movements to targets, which were visible the whole time, participants adapted to the task in AEP, AIP, and CP. Effector-dependent representations of the sequence were acquired in AEP, but not in AIP. Even after extensive practice, effector-independent intrinsic representations of the sequence were not acquired. Effector-independent visual-spatial representations were acquired in both AEP and AIP. The time course of the acquisition of effector-independent visual-spatial representations did not differ between AEP and AIP. The present results indicate that AIP is a useful tool to acquire visual-spatial representations of actions. However, to acquire effector-dependent motor representations of actions, AIP may not replace AEP.

## Supplementary Material

Supporting Information

## Figures and Tables

**Table 1:** Overview of the learning and representation types together with the corresponding comparisons

Learning type	Comparisons
Sequence-unspecific learning	Different sequence in the practice hand: pretest vs. posttest and/or different sequence in the practice hand: experimental group vs. control group
Sequence-specific learning	Practice hand: practice sequence vs. different sequence and/or practice sequence in the practice hand: experimental group vs. control group
Representation type	Comparisons
Effector-dependent representations	Practice sequence: practice hand vs. transfer hand and/or mirror sequence: practice hand vs. transfer hand
Effector-independent intrinsic representations	Transfer hand: mirror sequence vs. different sequence and/or mirror sequence in the transfer hand: experimental group vs. control group
Visual-spatial representations	Transfer hand: practice sequence vs. different sequence and/or practice sequence in the transfer hand: experimental group vs. control group
abstract representations	Practice hand: mirror sequence vs. different sequence and/or mirror sequence in the practice hand: experimental group vs. control group

Note. For each type of learning and each type of representation statistical significance in one of the outlined comparisons is sufficient to indicate such learning or such a representation

**Table 2:** Sociodemographic data of the action-imagery practice, action-execution practice, and control-practice group

	Action-imagery practice	Action-execution practice	Control-practice	*p*
Sex, *N*_female_/*N*_male_	28/22	29/25	31/18	0.597
Age, *M* ± SD	24.9 ± 3.9	24.7 ± 4.1	24.5 ± 3.8	0.899
Laterality index, *M* ± SD	93.1 ± 12.1	95.1 ± 9	92.9 ± 10.1	0.493
External visual imagery, *M* ± SD	1.7 ± 0.6	2 ± 0.7	2 ± 0.7	0.079
Internal visual imagery, *M* ± SD	1.6 ± 0.5	1.8 ± 0.7	1.7 ± 0.5	0.191
Kinesthetic imagery, *M* ± SD	1.8 ± 0.8	1.8 ± 0.8	2 ± 0.8	0.255

Note. To compare the practice groups, a *X*^2^ test was calculated for the distribution of sex and ANOVAs with the factor practice group (action-imagery, action-execution, control action) were computed for the remaining variables

**Table 3:** Statistical values of the ANOVA on movement times

	*F*	*df*1, *df*2	*p*	$\eta_{\text{p}}^{2}$
Practice group	2.9	2, 150	0.056	0.04
Hand	2.7	1, 150	0.103	0.02
Sequence	79.2	2, 300	< 0.001	0.35
Session	167.2	3.3, 493.9	< 0.001	0.53
Practice group × hand	0.1	2, 150	0.867	< 0.01
Practice group × sequence	21.3	4, 300	< 0.001	0.22
Practice group × session	3.9	6.6, 493.9	0.001	0.05
Hand × sequence	3.4	2, 295.5	0.035	0.02
Hand × session	0.4	3.6, 543.6	0.798	< 0.01
Sequence × session	5.3	7.1, 1067.5	< 0.001	0.03
Practice group × hand × sequence	2.3	3.9, 295.5	0.061	0.03
Practice group × hand × session	1.6	7.2, 543.6	0.134	0.02
Practice group × sequence × session	1.8	14.2, 1067.5	0.001	0.02
Hand × sequence × session	1.1	8, 1201.3	0.357	0.01
Practice group × hand × sequence × session	1.3	16, 1201.3	0.197	0.02

Note. The ANOVA was conducted with the factors practice group (action-imagery, action-execution, and control action), hand (practice, transfer), sequence (practice, mirror, different), and session (1, 2, 3, 4, 5, 6)

## Declarations

1 A detailed analysis of errors revealed no significant effects (see supplemental material).

2 The detailed analysis including the factor practice hand can be found in the supplemental material.
