# Supplementary material for: Sequence representations after action-imagery practice of one-finger movements are effector-independent
Source: Psychol Res. Author manuscript; Available in PMC 2023 Feb 1. (PMC9873765; doi:10.1007/s00426-022-01645-3)
Supplement: Supporting Information [file EMS144427-supplement-Supporting_Information.pdf]

SUPPLEMENTAL MATERIAL

Sequence representations after action imagery practice of one-finger movements are effector independent

Stephan F. Dahm [1], Matthias Weigelt [2] & Martina Rieger [1]

[1] UMIT - University for Health Sciences Medical Informatics and Technology

[2] University Paderborn

Overview

This document includes additional analyses and descriptive statistics that may be of interest to some readers. In a first step, we report the analysis that includes the transfer direction (from left to right hand or vice versa), which is otherwise only briefly mentioned in the main manuscript. In a second step, we report standard errors of movement times, which are not reported in the manuscript. In a third step, we report analyses of the error rates.

1. Analysis of the direction of intermanual transfer

To investigate the effects of transfer direction on movement times, we calculated a mixed-model ANOVA with the between factors practice group (AIP, AEP, CP) and practice hand (left, right) and the within factors test hand (practice, transfer), sequence (practice, mirror, different), and session (1, 2, 3, 4, 5, 6). Means of the movement times are shown in Figure SM1 and the corresponding standard errors in Table SM1. Results of the ANOVA are shown in Table SM2. Here, we focus on the main effect and interactions with the factor 'practice hand'.

The interaction between practice hand and test hand was significant. In the left-hand practice group, MTs were shorter in the right transfer hand (*M* = 4.8 s) than in the left practice hand (*M* = 5 s). In the right-hand practice group, MTs were shorter in the right practice hand (*M* = 5 s) than in the left transfer hand (*M* = 5.2 s). All remaining effects with the factor practice hand were not significant.

In short, participants performed faster with the right dominant hand than with the left non-dominant hand. Because this effect was already observed before any experimental manipulations in Session 1 and no significant further effects with the factor practice hand were observed, we decided to remove the factor practice hand from the analysis in the main manuscript.

Figure SM1. Means of movement times (in s) depending on test hand (practice, transfer), sequence (practice, mirror, different), and session (1, 2, 3, 4, 5, 6), separately for the practice groups (left-hand imagery practice, right-hand imagery practice, left-hand execution practice, right-hand execution practice, left-hand control practice, and right-hand control practice).

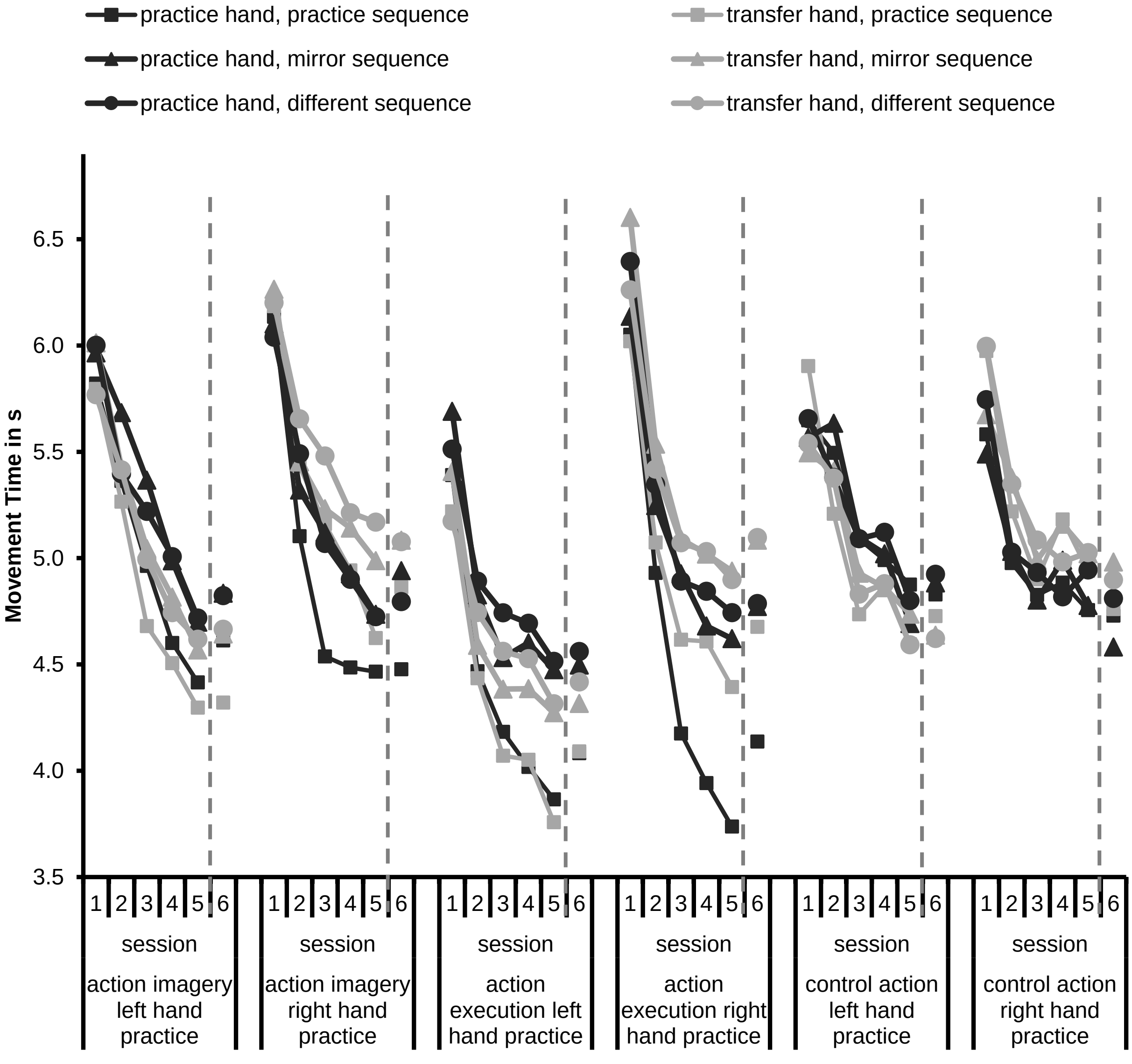

Table SM1. Standard errors of the means of movement times (in ms) depicted in Figure SM1 depending on session (1, 2, 3, 4, 5, 6), test hand (practice, transfer), and sequence (practice, mirror, different), separated for the practice groups (left-hand imagery practice, right-hand imagery practice, left-hand execution practice, right-hand execution practice, left-hand control practice, and right-hand control practice).

			Practice hand			Transfer hand		
			Practice sequence	Mirror sequence	Different sequence	Practice sequence	Mirror sequence	Different sequence
Action imagery practice	Left hand practice	Session 1	223	259	252	242	314	200
		Session 2	182	248	149	245	211	195
		Session 3	141	213	156	184	225	195
		Session 4	144	178	159	166	194	172
		Session 5	171	182	167	189	203	171
		Session 6	175	174	176	177	192	193
	Right hand practice	Session 1	234	255	262	301	241	295
		Session 2	262	211	248	218	262	247
		Session 3	159	238	213	198	258	244
		Session 4	155	246	230	158	220	201
		Session 5	139	188	157	149	180	214
		Session 6	135	179	148	141	193	163
Action exe-cution practice	Left hand practice	Session 1	225	282	195	197	193	141
		Session 2	123	172	154	132	130	155
		Session 3	116	136	175	122	122	127
		Session 4	142	149	148	169	158	150
		Session 5	131	125	167	99	166	147
		Session 6	142	145	126	143	135	128
	Right hand practice	Session 1	261	267	256	223	252	204
		Session 2	206	185	206	200	214	214
		Session 3	160	235	177	169	195	177
		Session 4	152	187	217	166	174	198
		Session 5	166	185	225	157	222	199
		Session 6	194	194	219	177	244	195
Control practice	Left hand practice	Session 1	232	230	246	277	337	237
		Session 2	255	278	256	224	299	318
		Session 3	222	163	180	212	230	251
		Session 4	204	211	212	204	223	216
		Session 5	213	148	193	194	212	171
		Session 6	155	185	177	149	181	163
	Right hand practice	Session 1	232	217	219	263	200	216
		Session 2	196	201	155	204	180	165
		Session 3	236	171	159	174	162	177
		Session 4	151	189	174	186	147	157
		Session 5	167	169	207	173	174	202
		Session 6	219	180	195	215	209	211

Table SM2. Statistical values of the ANOVA on MTs. Factors and factor levels were practice hand (left, right), practice group (action imagery practice, action execution practice, control practice), sequence (practice, mirror, different), test hand (practice, transfer), and session (1, 2, 3, 4, 5, 6). Only the main effect of and interactions with practice hand are shown.

	F	*df1, df2*	*p*	η^2_p
Practice hand	2.9	1, 147	.091	.02
Practice hand x Practice group	1.2	2, 147	.294	.02
Practice hand x Test hand	68.9	1, 147	<.001	.32
Practice hand x Sequence	1.9	2, 294	.158	.01
Practice hand x Session	2.3	3.4, 495.8	.066	.02
Practice hand x Practice group x Test hand	0.2	2, 147	.833	<.01
Practice hand x Practice group x Sequence	1	4, 294	.395	.01
Practice hand x Practice group x Session	1.4	6.7, 495.8	.193	.02
Practice hand x Test hand x Session	1.3	3.7, 543.3	.258	.01
Practice hand x Test hand x Sequence	0.3	2, 294	.725	<.01
Practice hand x Sequence x Session	0.7	7.2, 1057	.636	.01
Practice hand x Practice group x Test hand x Sequence	1.1	4, 294	.361	.02
Practice hand x Practice group x Test hand x Session	0.7	7.4, 543.3	.683	.01
Practice hand x Test hand x Sequence x Session	1.2	8.2, 1202.8	.293	.01
Practice hand x Practice group x Test hand x Sequence x Session	0.8	16.4, 1202.8	.673	.01

2. Standard errors of movement times in test blocks

Table SM3 shows the standard errors of the mean of movement times depicted in Figure 2 of the main manuscript, depending on practice group (action imagery practice, action execution practice, control practice), hand (practice, transfer), session (1, 2, 3, 4, 5, 6), and sequence (practice, mirror, different).

Table SM3. Standard errors of the mean of movement times (in ms) depending on session (1, 2, 3, 4, 5, 6), hand (practice, transfer), and sequence (practice, mirror, different), separately for the practice groups (action imagery practice, action execution practice, control practice).

		Practice hand			Transfer hand		
		Practice sequence	Mirror sequence	Different sequence	Practice sequence	Mirror sequence	Different sequence
Action imagery practice	Session 1	163	181	181	197	193	185
	Session 2	164	162	149	162	170	160
	Session 3	110	161	134	140	172	162
	Session 4	106	155	143	118	149	137
	Session 5	108	130	113	120	137	144
	Session 6	108	124	113	117	139	127
Action execution practice	Session 1	175	196	168	157	175	141
	Session 2	119	128	129	123	136	136
	Session 3	96	133	124	107	120	111
	Session 4	103	117	127	124	124	126
	Session 5	104	108	137	99	142	127
	Session 6	117	119	122	119	143	122
Control practice	Session 1	162	156	163	189	192	162
	Session 2	163	174	149	150	171	175
	Session 3	162	119	119	136	138	152
	Session 4	125	140	137	138	133	131
	Session 5	133	112	141	131	137	135
	Session 6	134	130	131	131	139	134

3. Standard errors of movement times during practice

Table SM4 shows the standard errors of the mean of movement times depicted in Figure 3 in the main manuscript, depending on practice group (action imagery practice, action execution practice, control practice) and session (1, 2, 3, 4).

Table SM3. Standard errors of the mean of movement times during practice (in ms) depending on session (1, 2, 3, 4, 5, 6) separately for the practice groups (action imagery practice, action execution practice, control practice).

	Action imagery practice	Action execution practice	Control practice
Session 1	135	130	137
Session 2	106	102	107
Session 3	103	99	104
Session 4	112	108	113

4. Error rates

To investigate the practice effects on error rates and potential speed-accuracy tradeoffs, we calculated a mixed-model ANOVA with the between factor practice group (AIP, AEP, CP) and the within factors hand (practice, transfer), sequence (practice, mirror, different), and session (1, 2, 3, 4, 5, 6). Means of the error rates are shown in Figure SM2 and the corresponding standard errors in Table SM4. Results of the ANOVA are shown in Table SM5. The ANOVA revealed no significant effects.

Figure SM2. Means of error rates (in %) depending on hand (practice, transfer), sequence (practice, mirror, different), and session (1, 2, 3, 4, 5, 6), separately for the practice groups (action imagery practice, action execution practice, control practice).

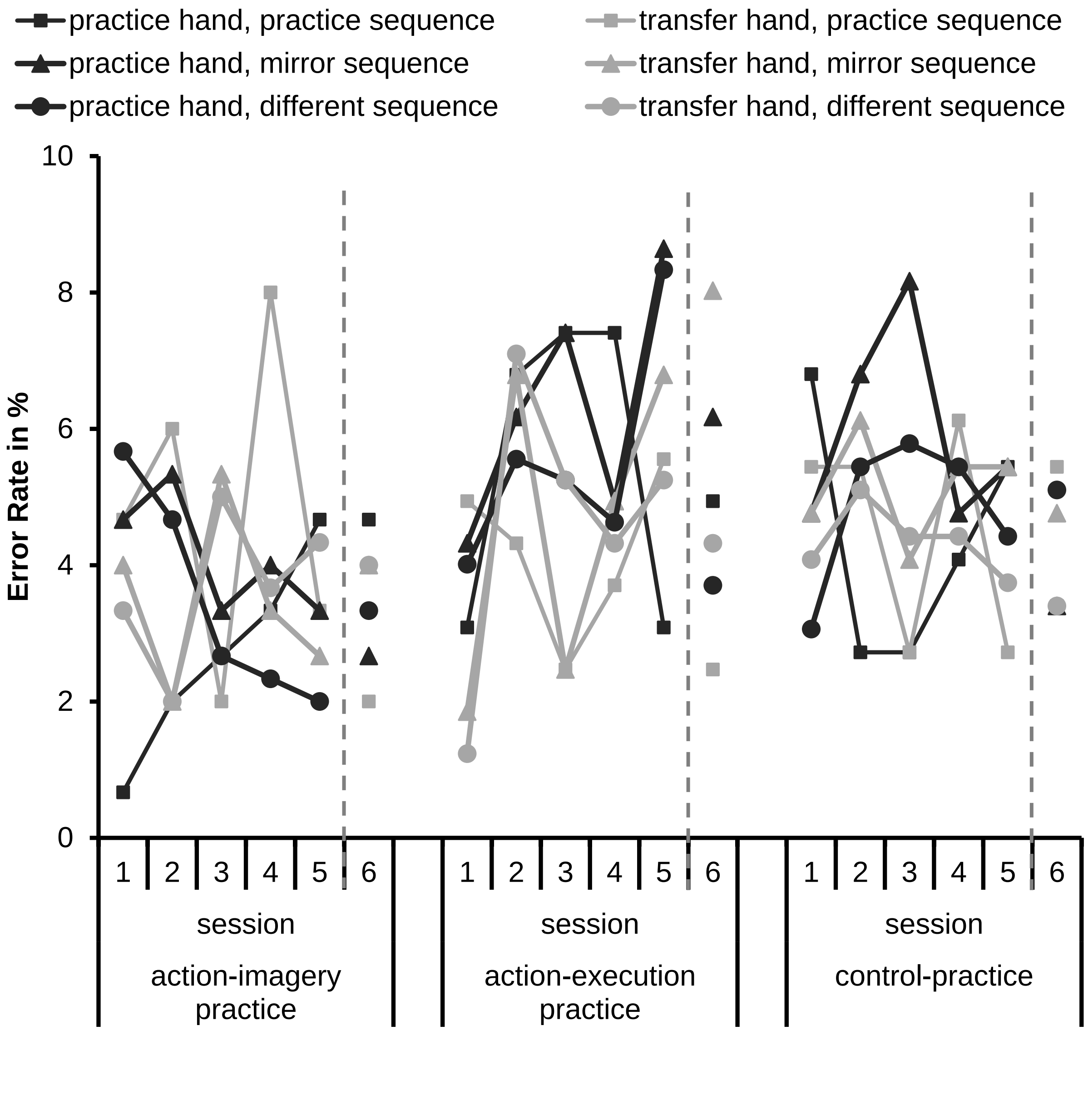

Table SM4. Standard errors of the mean of error rates (in %) depending on session (1, 2, 3, 4, 5, 6), hand (practice, transfer), and sequence (practice, mirror, different), separately for the practice groups (action imagery practice, action execution practice, control practice).

		Practice hand			Transfer hand		
		Practice sequence	Mirror sequence	Different sequence	Practice sequence	Mirror sequence	Different sequence
Action imagery practice	Session 1	0.67	1.65	1.69	1.91	1.55	1.06
	Session 2	1.13	1.99	1.26	2.27	1.13	1.03
	Session 3	1.29	1.72	0.99	1.13	1.99	1.37
	Session 4	1.43	1.55	0.83	2.03	1.43	1.19
	Session 5	1.91	1.43	0.77	1.43	1.29	1.04
	Session 6	1.91	1.29	1.06	1.13	1.55	1.22
Action execution practice	Session 1	1.33	1.54	0.98	1.85	1.05	0.60
	Session 2	2.04	1.98	1.32	1.54	1.84	1.43
	Session 3	1.90	2.10	1.31	1.20	1.20	1.38
	Session 4	2.10	1.63	1.20	1.69	1.85	1.26
	Session 5	1.33	2.19	1.80	1.92	1.84	1.15
	Session 6	1.85	1.78	1.05	1.20	2.32	1.10
Control practice	Session 1	1.94	1.94	1.05	1.78	1.68	1.24
	Session 2	1.32	2.17	1.13	1.78	1.86	1.30
	Session 3	1.32	2.29	1.50	1.32	1.58	1.27
	Session 4	1.85	1.94	1.49	1.86	2.03	1.17
	Session 5	1.78	1.78	1.27	1.32	1.78	1.00
	Session 6	1.46	1.46	1.30	2.03	1.68	1.08

Table SM5. Statistical values of the ANOVA on error rates. Factors and factor levels were practice group (action imagery practice, action execution practice, control practice), sequence (practice, mirror, different), hand (practice, transfer), and session (1, 2, 3, 4, 5, 6).

	F	$df1, df2$	p	η^2_p
Practice group	3	2, 150	.051	.04
Hand	1	1, 150	.328	.01
Sequence	1.8	1.9, 287.5	.169	.01
Session	1.1	5, 750	.348	.01
Practice group x Hand	2.1	2, 150	.125	.03
Practice group x Sequence	0.3	3.8, 287.5	.840	.01
Practice group x Session	1.2	10, 750	.286	.02
Hand x Sequence	0.6	1.8, 277.3	.561	<.01
Hand x Session	0.7	5, 750	.597	.01
Sequence x Session	0.8	9.5, 1418.7	.576	.01
Practice group x Hand x Sequence	0.6	3.7, 277.3	.635	.01
Practice group x Hand x Session	0.8	10, 750	.613	.01
Practice group x Sequence x Session	0.9	18.9, 1418.7	.529	.01
Hand x Sequence x Session	1	9.2, 1374.5	.467	.01
Practice group x Hand x Sequence x Session	1.5	18.3, 1374.5	.073	.02
